# The Mental Functions of the Brain

**Published:** 1902-04

**Authors:** 


THE MENTAL FUNCTIONS OF THE BRAIN. An Investigation into their Localisation and their Manifestation in Health and Disease. By BERNARD HOLLANDER, M. D., M. R. C. S., L. R. C. P. (London). Illustrated with clinical records of 800 cases of localised brain derangements and with several plates. Octavo, Pp. 523. New York and London: G. P. Putnam's Sons. 1901.

The study of the mental functions of the brain has not made such rapid advancements during the past twenty years as some of the collateral sciences, and deviations from the normal mental state remain little understood and far remote from cure. With this defect in knowledge in mind the author aims in the present work to clear up the mystery of the fundamental psychical functions and their localisation in the brain. The author claims for this work the distinction of being the first work on the subject since the dawn of modern scientific research. This is perhaps a doubtful distinction, as to many it might seem to be only an attempt and surely other attempts have been made since the new era in science began (date not given.)

The author further states that brain surgery should, if future investigators confirm the author's observations, receive an immense stimulus to activity, and the data amassed by the author, and published in this work are so considerable as to open up quite a new field for research.

The author found that his localisations confirm those made a century ago by Gall, "whose marvelous discoveries of the anatomy and physiology of the brain, on which Spurzheim built his system of phrenology, were ignored even by his most scientific followers, so that the world is ignorant of them, and they are presented for the first time in this book." "No subject has been so thoroughly misrepresented, even by learned men of acknowledged authority, and no author has ever been so libeled and with such malice as Gall and this notwithstanding the fact that there is not one man of scientific repute who has written anything which would indicate that he has examined Gall's chief work, Anatomie et Physiologie du Systeme Neveux."

This quotation from the preface of the book shows what the contents might be and they fairly represent the ideas or Gall's ideas rejuvenated by Hollander. Science is not jealous of any man, and any man who does honest research work is respected by scientific men the world over. There is no need at this late day of any champion in Gall's behalf, as his fate is as securely sealed as is that of Paracelsus or Mesmer, and no effort of Hollander's can unwind the thread of destiny that has been wound all one way for the past ten decades about the name and work of Gall or Spurzheim. This work is an interesting one to read, but not a wise one to follow, unless one is willing to surrender the ideas of Meynert, Charcot, Waldeyer, Bastian, Spitzka, Wilder and a host of other men of good repute. While there is much to learn and to respect regarding the configuration of the head—and while we are willing to go with Benedikt a long way on his travels, we hesitate to go into the jungles where Gall and Spurzheim would lead us because of the uncertainty of safe return to a scientific standpoint. The phrenology of Gall and Spurzheim is not the phrenology of scientific minds any more than the hypnotic exhibitions of Mesmer are the scientific unravelings of Charcot or Bernheim.

The greater part of this book is taken up with the defense of Gall's discoveries and some of the conclusions arrived at are as follows:

> Morbid fear and melancholia are connected with the supramarginal and angular gyri of the parietal lobe. Kleptomania and voracious hunger and abnormal thirst with the anterior part of the temporal lobes, superior and inferior respectively. Irascibility and violent mania with the central part of the temporal lobes. Mania of suspicion and persecution with the posterior part of the temporal lobes. The tender domestic and social affections with the occipital lobe. Perception of tone (music) and of numbers with some part of the brain abutting on the fissure of Sylvius. Perception of form, size, place, color, memory of time, facts and events with the supraorbital gyri and adjoining parts of the frontal convolutions. Imagination and co-ordinated processes with the antero-posterior part of the frontal lobes. Religious mania and perversion of altruistic sentiments with the posterior-superior part of the frontal lobes. Satyriasis and nymphomania with the hemispheres of the cerebellum.

And then this sage advice:

> Chief of all, however, the book should enable the physician or surgeon when he meets with cases in which the chief or perhaps the only symptoms are psychical not physical, to localise the seat of disease and to apply treatment accordingly. A new sphere is thus opened up for the practice of our profession —to the physicians of the mind as well as to the body.

If this be all true, then every page of this book deserves to be mounted in gold and hung in the consultation room of every physician the world over. If it is not true, and the leading scientific men of the world from Gall's time to the present day say *not*, then the book should be placed on the library shelf as one of those volumes which amuse rather than edify.

